# Effects of transcutaneous auricular vagus nerve stimulation combined with repetitive facilitative exercise on lower limb motor function in patients with intracerebral hemorrhage: A pilot randomized controlled trial

**DOI:** 10.1371/journal.pone.0352321

**Published:** 2026-06-30

**Authors:** Hanji Chen, Hanbo Chen, Chongrui Feng, Jiafa Liu, Xiaoli Li, Gengbiao Zhang, Zhanhao Liu, Mengyun Li

**Affiliations:** Guangdong Sanjiu Brain Hospital, Guangzhou, Guangdong, China; TIU: Tishk International University, IRAQ

## Abstract

**Objective:**

To evaluate the clinical efficacy of transcutaneous auricular vagus nerve stimulation (taVNS) combined with repetitive facilitative exercise (RFE) in improving lower limb function in patients with intracerebral hemorrhage.

**Methods:**

The research protocol was registered on the Chinese Clinical Trial Registry website (ChiCTR2500106064). Sixty patients with intracerebral hemorrhage were randomly assigned to either the experimental group (n = 30) or the control group (n = 30). The experimental group received real taVNS combined with RFE, while the control group received sham taVNS combined with RFE. Before and after 6 weeks of treatment, functional assessments were conducted using the Fugl-Meyer Assessment-Lower Extremities (FMA-LE), the Functional Ambulation Category (FAC) scale, and a three-dimensional gait analysis system to obtain kinematic parameters of the pelvis during the walking cycle.

**Results:**

Baseline demographic and clinical characteristics showed no significant differences between the two groups (all P > 0.05). After 6 weeks of intervention, both groups exhibited significant improvements in FMA-LE, FAC, stride length, peak hip extension angle, and peak hip joint moments (all P < 0.05). However, the experimental group demonstrated superior outcomes compared to the control group in FMA-LE (t = 3.233, P = 0.002), FAC (t = 2.868, P = 0.006), stride length (t = 3.077, P = 0.003), and peak hip extension angle (t = 2.682, P = 0.010). No significant difference was observed in peak hip joint moments (t = −0.212, P = 0.833) between the two groups after treatment. No adverse effects or dropouts were reported during the study.

**Conclusion:**

Compared to sham taVNS combined with RFE, taVNS combined with RFE can significantly enhances lower limb motor function in patients with post-stroke hemiplegia following intracerebral hemorrhage.

The research protocol has been registered on the Chinese Clinical Trial Registry website (ChiCTR2500106064).

## Introduction

Intracerebral hemorrhage (ICH), one of the most severe subtypes of stroke, imposes a substantial burden on patients and society due to its high disability and mortality rates. More than half of ICH patients experience varying degrees of limb motor dysfunction after the acute phase, with lower limb dysfunction being particularly prominent. This manifests as muscle weakness, abnormal muscle tone, loss of motor control, and balance impairments [1,2], which severely restrict walking ability and daily living, significantly increase fall risk, and ultimately lead to a substantial decline in quality of life and social participation [3].

The pathophysiological mechanisms underlying post-ICH motor dysfunction involve both primary brain injury and secondary neurological damage. The mass effect of hematoma and blood degradation products damage motor pathways such as the corticospinal tract, while secondary inflammatory responses and oxidative stress exacerbate neuronal apoptosis and disrupt neural network integrity [1,4,5]. Therefore, developing interventions to promote neural remodeling and reconstruct motor pathways remains a critical challenge in ICH rehabilitation.

Repetitive Facilitative Exercise (RFE), also known as the Kawakita method, is an innovative rehabilitation approach combining task-oriented training with peripheral nerve electrical stimulation [6]. By employing high-frequency repetitive movement training to enhance sensorimotor feedback and electrical stimulation to increase spinal α-motor neuron excitability, RFE facilitates the reconstruction of damaged neural pathways [7]. Clinical studies have demonstrated that RFE significantly improves limb motor function in stroke patients, particularly in upper limb recovery, with notable therapeutic efficacy [8,9].

Transcutaneous auricular vagus nerve stimulation (taVNS), a non-invasive neuromodulation technique, has introduced new therapeutic options for stroke rehabilitation. By activating the vagus nerve, a key pathway connecting the brainstem to peripheral organs, taVNS enhances noradrenergic neuron activity, promotes brain-derived neurotrophic factor release, and improves synaptic plasticity and neuronal survival [10,11]. Moreover, taVNS modulates autonomic nervous system function, suppresses excessive inflammation, and increases peri-lesional cerebral blood flow, creating a favorable microenvironment for neural repair [12,13]. Recent randomized controlled trials indicate that taVNS combined with conventional rehabilitation significantly enhances motor recovery in chronic stroke patients, likely due to cortical excitability rebalancing and motor network reorganization [14].

Building upon these advances, this study proposes an innovative rehabilitation strategy combining taVNS and RFE to synergistically improve motor dysfunction in ICH patients through central neuromodulation and peripheral facilitation, targeting multiple levels of neural recovery. However, clinical studies on the combined application of these two techniques remain scarce, and their efficacy and mechanisms require further validation. Therefore, this study will systematically investigate the effects of taVNS combined with RFE on lower limb motor function in ICH patients, providing new evidence-based insights for ICH rehabilitation.

## Patients and methods

### Study design

A prospective, single-blinded, sham-controlled, single-center clinical trial was conducted. In this study, allocation concealment was implemented using the sealed envelope method. An independent statistician generated the randomization sequence by computer (GraphPad Software) and placed the allocation results into sequentially numbered, opaque, sealed envelopes, which were then kept by a third party. When participants were enrolled, researchers opened the envelopes in sequential order to obtain the allocation results. To verify allocation concealment, we confirmed that all envelopes remained intact and were opened in the correct order; no concealment breaches were identified. In this study, the patients were blinded to their group assignment (active taVNS vs. sham taVNS).

**Fig 1:**
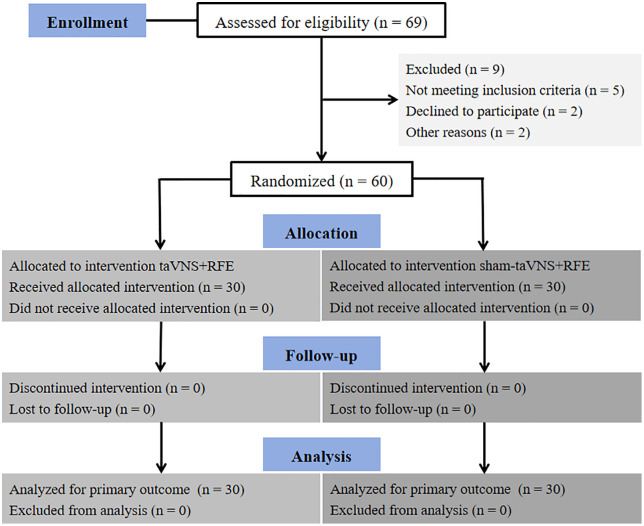
Flowchart of the study. taVNS: transcutaneous auricular vagus nerve stimulation; RFE: repetitive facilitative exercise.

This study adhered to the principles of the Declaration of Helsinki and ethical guidelines for human research, with approval obtained from the Ethics Committee of the Guangdong Sanjiu Brain Hospital (NO. 2023-01-022). However, due to an administrative delay between ethical approval and the formal issuance of the approval document, the trial registration was completed after patient enrollment had begun. We confirm that all ongoing and related trials for this intervention are registered on the Chinese Clinical Trial Registry website (ChiCTR2500106064). Written informed consent was obtained from all participants prior to their inclusion in the study. The flowchart of the study was shown in Fig 1.

### Patients

Sample size calculation: Using the formula for comparing means between two independent samples, the calculation was conducted with PASS 15.0 software. The effect size was set at d = 0.8 based on the minimal clinically important difference for the Fugl-Meyer Assessment Lower Extremity (FMA-LE), which has been established as 6 points in chronic stroke patients [15]. With a significance level of α = 0.05 (two-tailed test) and a power of 1-β = 0.8, a minimum of 26 participants per group was required. Accounting for a 10% dropout rate, at least 29 patients needed to be enrolled in each group.

All ICH patients were recruited from the Department of Rehabilitation Medicine, Guangdong Sanjiu Brain Hospital between 01/01/2025 and 30/08/2025, and follow-up was completed before 30/10/2025. The inclusion criteria were as follows: (1) non-brainstem hemorrhage confirmed by cranial MRI or CT; (2) stable condition with a disease duration of 1–3 months; (3) age > 18 years; (4) can walk independently or with assistance. Exclusion criteria included: (1) moderate to severe cognitive impairment (the Montreal Cognitive Assessment score ≤ 18) preventing compliance with treatment; (2) contraindications for auricular stimulation, such as wounds or infections in the ear; (3) severe systemic impairments or complications involving the heart, liver, or kidneys; (4) lower limb fractures, joint stiffness, or muscle spasms; and (5) presence of a cardiac pacemaker or cochlear implant.

### Intervention protocol

The experimental group received taVNS combined with RFE, while the control group received sham taVNS combined with RFE. The intervention sequence involved simultaneous administration of RFE and taVNS. Both groups continued to receive standard pharmacological treatment and conventional rehabilitation therapy.

### Repetitive facilitative exercise

All therapeutic procedures were performed by certified senior therapists trained in the Kawashima method. The treatment followed a systematic four-step approach. First, with the patient in a supine position with knees flexed, the therapist alternately tapped and brushed the hip external rotator/abductor and internal rotator/adductor muscle groups while guiding corresponding hip abduction/extension and flexion/adduction movements. Second, maintaining the supine position, the therapist stimulated the hip abductors and adductors to induce compound movements including hip extension, abduction, external rotation, and knee extension. Third, in the side-lying position, the therapist applied brushing stimuli to the hip flexor/extensor muscles while instructing hip flexion/extension exercises. Finally, the therapist simultaneously stimulated the healthy-side gluteus medius and affected-side inguinal ligament while guiding stepping movements of the affected limb, with fall prevention measures throughout. Each patient performed 100 standardized repetitions per movement daily. Each training session lasted approximately 40 minutes, and treatment was delivered 5 days per week for 6 weeks to ensure therapeutic efficacy.

### Transcutaneous auricular vagus nerve stimulation

**Fig 2:**
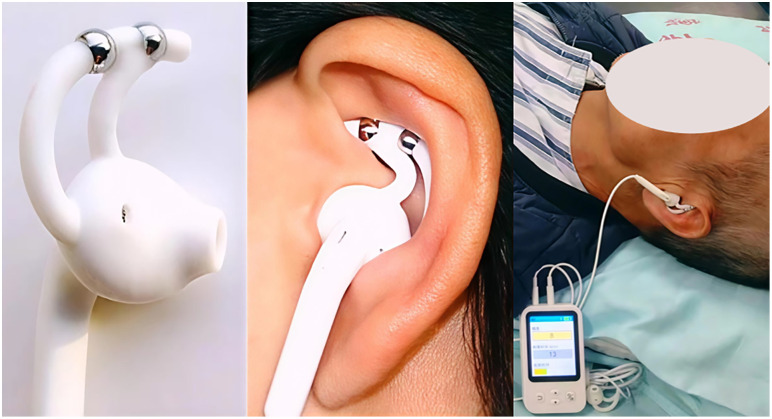
Schematic diagram of taVNS stimulation.

taVNS was administered using a low-frequency stimulator (RISHENA tVNS501). The protocol consisted of 25 Hz stimulation at 500μs pulse width in an intermittent mode (30s on/30s off), delivered in 30-minute sessions 5 days per week for 6 weeks (30 sessions total). The current amplitude was individually set within a range of 0.1–5.0 mA, adjusted to each participant’s sensory threshold without inducing pain. Standardized procedures included: pretreatment disinfection of the left concha (Fig 2) and electrodes with 75% alcohol swabs; precise electrode placement in the left concha with medical tape reinforcement if needed; and confirmation of proper “connection status” (blue-green indicator) before stimulation. Therapists monitored patients throughout to optimize electrode positioning and stimulation effectiveness. The left concha was chosen for taVNS based on safety concerns and the high density of vagus nerve innervation in this area [16,17].

In the sham stimulation protocol, the current intensity threshold was assessed at the beginning of each session, after which the current intensity was gradually ramped down to zero during the RFE therapy. This approach allowed patients in the sham group to experience the sensation of the treatment procedure without receiving stimulation sufficient to produce a therapeutic effect [18].

### Outcome measures

The primary outcome of this study was the Fugl-Meyer Assessment for Lower Extremities (FMA-LE), which was used to comprehensively evaluate the recovery of lower limb motor function. Secondary outcomes included the Holden Functional Ambulation Classification (FAC) scale and key kinetic parameters of the hemiplegic side gait measured precisely by a three-dimensional gait analysis system (SMART-DX400), including peak hip extension angle, peak hip joint moments, and stride length, which were used to assess walking ability and pelvic and lower limb motor control. Data were collected from all patients at pre-treatment and after 6 weeks of treatment. Any adverse events occurring during the study period, such as skin irritation, dizziness, discomfort, or falls, will be recorded and analyzed for their relationship to the interventions.

### Statistical analysis

Statistical analyses were performed using SPSS 26.0 software (IBM SPSS Inc., Chicago, IL, USA). Normality tests were first conducted for continuous variables, with data presented as mean ± standard deviation (M ± SD) or median (interquartile range) [M(IQR)] depending on distribution. Baseline characteristics and post-intervention outcomes were analyzed using chi-square tests, independent samples t-tests, or Mann-Whitney U tests as appropriate. Within-group comparisons (pre- vs post-intervention) for both experimental and control groups were performed using paired t-tests or nonparametric tests. Statistical significance was set at p < 0.05.

## Results

### Demographic characteristics

A total of 60 patients with cerebral hemorrhage were enrolled in this study and randomly allocated into two groups, with 30 cases in each group. All participants successfully completed the clinical trial without any case dropout or adverse reactions. There were no statistically significant differences in baseline characteristics between the two groups, including gender distribution, mean age, body mass index (BMI), disease duration, and hemiplegic side (p > 0.05), indicating good comparability (Table 1).

**Table 1:** Baseline demographic and clinical characteristics of the patients.

Variable	Experimental N = 30	Control N = 30	Tests statistic	P
Gender (male/ female)	18/ 12	21/ 9	0.659	0.589
Age (years)	59.90 ± 9.51	61.20 ± 8.95	−0.545	0.588
BMI (kg/m^2^)	21.17 ± 1.57	21.44 ± 1.47	−0.680	0.499
Hemiplegic side (left/right)	14/ 16	12/ 18	0.271	0.795
Disease duration (day)	45.19 ± 12.98	44.14 ± 10.26	0.348	0.729
FMA-LE	12.60 ± 1.30	12.37 ± 1.19	0.725	0.471
FAC	1.90 ± 0.80	1.77 ± 0.73	0.674	0.503
Peak hip extension angle (°)	7.05 ± 1.80	6.67 ± 1.28	0.951	0.346
Peak hip joint moments (Nm/kg)	0.27 ± 0.10	0.27 ± 0.10	0.064	0.949
Stride length (m)	0.22 ± 0.06	0.21 ± 0.07	0.722	0.473

BMI: Body mass index; FMA-LE: lower extremity subscale of the Fugl-Meyer Assessment; FAC: Functional Ambulation Classification.

### Lower limb motor function

At baseline, there were no significant differences between the two groups in FMA-LE, FAC, peak hip extension angle, peak hip joint moments (maximum and minimum), or stride length (P > 0.05) (Table 1). After 6 weeks of intervention, both groups showed significant improvements in these outcomes compared to baseline (P < 0.05). Moreover, the intervention group demonstrated superior outcomes in FMA-LE, FAC, peak hip extension angle, peak hip joint moments, and stride length compared to the control group (P < 0.05) (Tables 2 and 3).

**Table 2:** Comparison of lower limb motor function within-group comparisons.

Variable/Group	Pre- intervention	Post- intervention	95% CI	Tests statistic	P
**FMA-LE**					
Experimental	12.60 ± 1.30	22.47 ± 3.14	9.87 (8.97 to 10.77)	−24.468	***<0.001***
Control	12.37 ± 1.19	20.07 ± 2.59	7.70 (6.91 to 8.49)	−22.028	***<0.001***
**FAC**					
Experimental	1.90 ± 0.80	3.47 ± 0.68	1.57 (1.37 to 1.77)	−17.026	***<0.001***
Control	1.77 ± 0.73	2.97 ± 0.67	1.20 (1.04 to 1.36)	−16.155	***<0.001***
**Peak hip extension angle (°)**					
Experimental	7.05 ± 1.80	12.58 ± 1.91	5.53 (4.92 to 6.14)	−19.107	***<0.001***
Control	6.67 ± 1.28	11.28 ± 1.84	4.61 (4.18 to 5.04)	−24.324	***<0.001***
**Peak hip joint moments (Nm/kg)**					
Experimental	0.27 ± 0.10	0.30 ± 0.13	0.03 (0.00 to 0.06)	−2.101	***0.044***
Control	0.27 ± 0.10	0.31 ± 0.15	0.04 (0.01 to 0.07)	−2.284	***0.030***
**Stride length (m)**					
Experimental	0.22 ± 0.06	0.42 ± 0.06	0.20 (0.18 to 0.22)	−22.222	***<0.001***
Control	0.21 ± 0.07	0.38 ± 0.04	0.17 (0.15 to 0.19)	−18.889	***<0.001***

FMA-LE: lower extremity subscale of the Fugl-Meyer Assessment; FAC: Functional Ambulation Classification; CI: confidence interval.

**Table 3:** Comparison of lower limb motor function between-group comparisons (post-intervention).

Variable	Experimental Group	Control Group	95% CI	d	Tests statistic	P
**FMA-LE**	22.47 ± 3.14	20.07 ± 2.59	2.40 (0.91 to 3.89)	0.82	3.233	***0.002***
**FAC**	3.47 ± 0.68	2.97 ± 0.67	0.50 (0.15 to 0.85)	0.74	2.868	***0.006***
**Peak hip extension angle (°)**	12.58 ± 1.91	11.28 ± 1.84	1.30 (0.33 to 2.27)	0.69	2.682	***0.010***
**Peak hip joint moments (Nm/kg)**	0.30 ± 0.13	0.31 ± 0.15	−0.01 (−0.08 to 0.06)	0.07	−0.212	0.833
**Stride length (m)**	0.42 ± 0.06	0.38 ± 0.04	0.04 (0.01 to 0.07)	0.78	3.077	***0.003***

FMA-LE: lower extremity subscale of the Fugl-Meyer Assessment; FAC: Functional Ambulation Classification; CI: confidence interval.

## Discussion

Post-stroke hemiplegia following ICH is a common clinical impairment, and functional recovery is closely associated with neural remodeling. Hematoma compression and secondary injury disrupts motor pathways, leading to abnormal movement patterns that severely affect activities of daily living and impose a substantial burden on families and society [2,3]. Identifying effective interventions to promote neural remodeling and restore normal motor patterns remains a key research focus [19]. Based on neuroplasticity theory, this study combines taVNS with RFE as a novel therapeutic approach for lower limb dysfunction after ICH.

RFE facilitates functional reorganization of the central nervous system through specific peripheral sensory inputs [7]. Clinically, it improves pelvic control and lower limb motor function. The pelvis, as a critical junction between the trunk and lower limbs, directly influences gait quality. When ICH damages motor pathways, trunk control and core stability are often compromised, which directly affects pelvic coordination, manifesting as compensatory tilting or rotation during walking [20]. RFE utilizes high-frequency, rhythmic peripheral stimulation to activate spinal reflex pathways, enhance motor neuron pool excitability, and promote relearning of normal movement patterns [21,22].

As a non-invasive neuromodulation technique, taVNS exerts broader effects. The vagus nerve connects extensively with multiple brain regions via the nucleus tractus solitarius [23,24]. Recent studies suggest that electrical stimulation of the auricular concha not only activates the brainstem reticular formation but also modulates higher brain regions, including the prefrontal and sensorimotor cortices, creating a favorable neural environment for post-stroke recovery [25,26]. Notably, taVNS enhances neuroplasticity while also exhibiting anti-inflammatory and neuroprotective effects. During the acute phase of ICH, it mitigates cerebral edema and preserves blood-brain barrier integrity via cholinergic anti-inflammatory pathways [27,28]. During recovery, it facilitates functional reorganization by promoting neurotransmitter release and synaptic plasticity [10,29,30]. These multi-target effects make taVNS an ideal intervention for stroke rehabilitation.

This study integrates these two approaches into a proposed synergistic “central-peripheral” modulation model. We hypothesize that taVNS optimizes central plasticity from a top-down perspective, while RFE reinforces proprioceptive feedback and motor learning from a bottom-up approach [10,22,29]. This hypothesized bidirectional mechanism could potentially explain the observed superior clinical outcomes. The combined intervention group showed greater functional improvements, particularly in gait parameters such as peak hip torque and step symmetry, which are key indicators closely related to walking ability and clinical significance. However, peak hip joint moments did not show a significant between-group difference. Unlike kinematic parameters, joint moment is a kinetic measure reflecting muscle force generation, which may require more prolonged training [31]. Additionally, the 6-week intervention period may have been insufficient to produce measurable improvements in muscle strength [32]. Given the absence of direct neurophysiological or neuroimaging data, this mechanistic interpretation remains a hypothesis requiring future validation.

From a clinical application perspective, this combined regimen offers several advantages. First, the taVNS protocol is safe and well tolerated, with minimal adverse events observed in our study. Second, its operation is relatively straightforward and does not require complex equipment, facilitating adoption across diverse clinical settings. Third, by addressing the multi-level pathological mechanisms underlying post-ICH dysfunction, this strategy enables comprehensive functional rehabilitation. Despite the promising findings, the conclusion that the combined therapy is superior should be interpreted with caution and larger multicenter trials with extended follow‑up are warranted to confirm these effects.

### Limitation

This study has several limitations. First, as a single‑center randomized controlled trial, the generalizability of the conclusions may be limited. The patient population, diagnostic and treatment standards, and rehabilitation protocols of a single institution may not fully represent broader populations, which could affect the external validity of the findings. Second, the sample size was insufficient for subgroup analyses based on ICH features (e.g., location, volume). Third, the short follow-up period precludes assessment of long-term durability. Fourth, the lack of neurophysiological or neuroimaging data limits mechanistic interpretation. Consequently, the interpretation of therapeutic effects remains largely at the level of functional association. Furthemore, therapists were not blinded due to the nature of the intervention, introducing potential performance bias.

Future research should focus on the following directions. First, prospective, multicenter, large-sample randomized controlled trials should be conducted with patients stratified by key clinical and imaging characteristics to clarify the differential efficacy of this combined regimen across subgroups. Second, multimodal neuroimaging and neurophysiological monitoring techniques should be incorporated to longitudinally track changes in brain network reorganization, thereby providing mechanistic evidence for the observed benefits. Third, parametric studies should systematically explore optimal taVNS stimulation parameters and compare response patterns across patients in the acute, subacute, and chronic recovery phases, with the aim of establishing individualized, stage-adapted rehabilitation protocols. Finally, long-term follow-up assessments are needed to determine whether the observed improvements can be sustained over time.

## Conclusion

This study demonstrates that taVNS combined with RFE significantly improves lower limb motor function and gait ability in hemiplegic patients after ICH. Following six weeks of combined intervention, the experimental group exhibited superior outcomes in FMA-LE, FAC, and 3D gait parameters (step length and peak hip torque) compared to RFE alone. The underlying mechanism likely involves central-peripheral synergistic modulation. This combined approach is simple, safe, and offers a promising new strategy for post-ICH lower limb rehabilitation. Future research should expand sample sizes, incorporate long-term follow-up assessments, and utilize multimodal evaluations to further validate long-term efficacy and neural mechanisms.

## Supporting information

S1 Data CONSORT 2025 checklist of information to include when reporting a randomised trial. (DOCX)

S2 Data Study protocol. (PDF)
