# Supplementary material for: Effects of transcutaneous auricular vagus nerve stimulation combined with repetitive facilitative exercise on lower limb motor function in patients with intracerebral hemorrhage: A pilot randomized controlled trial
Source: PLoS One. 2026 Jun 30;21(6):e0352321. doi: 10.1371/journal.pone.0352321 (PMC13318024; doi:10.1371/journal.pone.0352321)
Supplement: S2 Data — (PDF) [file pone.0352321.s002.pdf]

Protocol for a Randomized Controlled Trial: Effects of Transcutaneous Auricular Vagus Nerve Stimulation Combined with Repetitive Facilitative Exercise on Lower Limb Motor Function in Patients with Intracerebral Hemorrhage

Project Manager: Hanbo Chen, Hanji Chen

Principal Investigator: Chongrui Feng, Zhanhao Liu, Mengyun Li, Jiafa Liu, Xiaoli Li, Gengbiao Zhang

1. Background and Objectives

Intracerebral hemorrhage (ICH) is one of the major stroke subtypes leading to severe disability and mortality. After the acute phase, patients often experience significant lower limb motor dysfunction, manifesting as muscle weakness, abnormal muscle tone, impaired coordination and motor control, and balance deficits. These functional impairments not only severely affect the patient's ability to walk independently and increase the risk of falls but also greatly limit their participation in daily activities and social functions, resulting in a marked decline in overall quality of life.

Currently, the recovery of motor function after ICH depends on the remodeling and compensation of the central nervous system. Promoting neuroplasticity has become a core goal of rehabilitation therapy. Repetitive Facilitative Exercise (RFE) is a rehabilitation technique that combines task-oriented repetitive movements with specific sensory input. It aims to reinforce correct movement patterns and reconstruct neural control pathways between the brain and limbs through high-intensity, repetitive functional task training supplemented by manual or instrumental facilitation of relevant muscle groups and nerves. Existing research suggests its potential in improving limb function after stroke.

On the other hand, non-invasive neuromodulation techniques offer new avenues for stroke rehabilitation. Transcutaneous Auricular Vagus Nerve Stimulation (taVNS) activates the vagus nerve pathway non-invasively by stimulating the auricular area where the vagus nerve distributes. This technique is believed to modulate the release of neurotransmitters (such as norepinephrine) in the brain, enhance levels of neurotrophic factors, and potentially optimize the microenvironment for neural repair by regulating neuroinflammation and cerebral blood flow, thereby promoting functional reorganization of the motor cortex and networks from the "central" side.

Theoretically, combining Repetitive Facilitative Exercise, which focuses on "peripheral facilitation" and "task repetition," with Transcutaneous Auricular Vagus Nerve

Stimulation, which focuses on "central modulation" and "neuroplasticity regulation," may produce synergistic effects. This combined intervention strategy is expected to simultaneously address both peripheral sensory input and central neuromodulation, promoting a more comprehensive recovery of lower limb motor function after ICH. However, there is a lack of high-quality clinical evidence regarding the combined application model and definitive efficacy of these two innovative techniques.

Therefore, this study aims to systematically evaluate the effects of Transcutaneous Auricular Vagus Nerve Stimulation combined with Repetitive Facilitative Exercise on lower limb motor function, walking ability, and gait parameters in patients during the recovery phase of ICH through a randomized controlled trial. The goal is to provide a new, evidence-based multimodal combined intervention strategy for ICH rehabilitation.

2. Research Methods

2.1 Study Design

This is a single-center, assessor-blinded, randomized controlled trial. Eligible patients will be randomly assigned to either the Combined Intervention Group (receiving real taVNS combined with RFE) or the Control Intervention Group (receiving sham taVNS combined with RFE). Both groups will receive consistent basic pharmacological treatment and other conventional rehabilitation therapies.

In this study, allocation concealment was implemented using the sealed envelope method. An independent statistician generated the randomization sequence by computer and placed the allocation results into sequentially numbered, opaque, sealed envelopes, which were then kept by a third party. When participants were enrolled, researchers opened the envelopes in sequential order to obtain the allocation results.

2.2 Study Participants

2.2.1 Sample Size Estimation: Based on preliminary trial results, using the sample size calculation formula for comparing means between two independent samples, with the primary outcome measure (FMA-LE score) expected effect size $d=0.8$, $\alpha=0.05$

(two-tailed), and power 1-β=0.8, an estimated 26 participants per group is required. Considering an approximate 10% dropout rate, 29 participants per group are planned, totaling 58 participants.

2.2.2 Inclusion Criteria:

1. Diagnosis of supratentorial ICH confirmed by CT or MRI, with stable vital signs and neurological function;
2. Disease duration of 1-6 months (recovery phase);
3. Age 18-75 years;
4. Presence of unilateral lower limb motor dysfunction (Brunnstrom stage of the affected lower limb ≥ III);
5. Ability to walk short distances with minimal assistance or supervision (Functional Ambulation Classification ≥ level 2);
6. Clear consciousness, able to understand and cooperate with assessments and treatments;
7. Signed informed consent from the patient or their legal guardian.

2.2.3 Exclusion Criteria:

1. Brainstem or cerebellar hemorrhage, or cerebrovascular diseases requiring surgical intervention;
2. Comorbid severe dysfunction of vital organs (e.g., heart, liver, kidneys) or systemic infections and other serious complications;
3. Orthopedic conditions affecting lower limb function (e.g., unhealed fractures, severe arthritis) or peripheral neuropathy;
4. Local skin lesions, infections, or eczema in the auricle, unsuitable for auricular electrical stimulation;
5. Implanted electronic medical devices such as cardiac pacemakers, defibrillators, deep brain stimulators, or cochlear implants;
6. History of epilepsy, psychiatric disorders, or severe cognitive impairment (MMSE

score < 21), unable to cooperate with the study;

7. Known or suspected allergy to electrode adhesive;

8. Concurrent participation in other clinical trials that may interfere with the results of this study.

2.3 Intervention Protocol

All interventions will be performed by uniformly trained therapists.

Combined Intervention Group: Receives Transcutaneous Auricular Vagus Nerve Stimulation synchronized with Repetitive Facilitative Exercise.

Control Intervention Group: Receives sham Transcutaneous Auricular Vagus Nerve Stimulation synchronized with Repetitive Facilitative Exercise.

The intervention frequency for both groups is 5 days per week for 6 weeks.

2.3.1 Repetitive Facilitative Exercise Protocol:

Therapists will select and perform facilitative exercises targeting key lower limb muscle groups and movement patterns based on the patient's functional level. The training will follow a standardized operational procedure, including:

1. Facilitation Induction Using specific techniques such as brushing, tapping, or vibration to stimulate target muscle groups or dermatomes to enhance their excitability.

2. Assisted Active Movement: Immediately after facilitation, the therapist assists the patient in attempting active movement of the target action, such as flexion/extension, adduction/abduction of the hip, knee, and ankle.

3. Repetitive Task Training: Integrating the elicited movements into functional tasks for high-repetition practice (e.g., 30-50 repetitions per set, multiple sets daily), such as bridging in supine, sit-to-stand transfers, weight shifting, and stepping exercises.

The training emphasizes movement quality, intention, and repetition, with gradual progression in difficulty and reduction of assistance based on patient improvement.

2.3.2 Transcutaneous Auricular Vagus Nerve Stimulation Protocol

Device and Parameters: A Transcutaneous Auricular Vagus Nerve Stimulator will be used.

Real stimulation parameters are set as: frequency 25Hz, pulse width 250-500μs, using an intermittent stimulation mode (e.g., 30 seconds on, 30 seconds off). Intensity is adjusted to a level where the patient feels a distinct but non-painful tingling sensation (typically 2-3mA below the perception threshold).

Stimulation Site: Electrodes are attached to the left concha area (distribution area of the auricular branch of the vagus nerve).

Sham Stimulation Setup: The device appearance, electrode placement location, and operational procedure are identical to the real stimulation group. However, the stimulator delivers an ineffective, very low-intensity current that is barely perceptible (e.g., <0.1mA) to mimic device operation.

2.4 Outcome Measures

Assessments will be conducted by professional rehabilitation therapists blinded to group allocation at baseline (pre-intervention) and immediately after the 6-week intervention period.

2.4.1 Primary Outcome Measure:

1.Lower Limb Motor Function: Assessed using the Fugl-Meyer Assessment for Lower Extremities.

2.4.2 Secondary Outcome Measures:

1. Walking Function: Assessed using the Functional Ambulation Classification.
2. Gait Analysis: Using a three-dimensional motion capture and force plate system to quantitatively analyze spatiotemporal parameters (e.g., gait speed, stride length, cadence) and kinematic parameters (e.g., angular changes of hip, knee, and ankle joints during the gait cycle) of the affected side.

2.5 Safety Monitoring

Any adverse events occurring during the study period, such as skin irritation, dizziness, discomfort, or falls, will be recorded and analyzed for their relationship to the interventions.

2.6 Statistical Analysis Methods

Data analysis will be performed using SPSS software. Continuous data conforming to a normal distribution will be presented as mean ± standard deviation and compared between groups using independent samples t-tests; within-group comparisons (pre- vs post-intervention) will use paired samples t-tests. Continuous data not conforming to a normal distribution will be presented as median (interquartile range) and analyzed using non-parametric tests. Categorical data will be analyzed using chi-square tests. All statistical tests will be two-sided, with a P-value < 0.05 considered statistically significant.

3. Quality Evaluation

3.1 Patient Compliance and Withdrawal

Establish a good relationship with subjects, provide health consultation, explain the benefits of participating in the experiment, and minimize dropouts or withdrawals during the study.

3.2 Protocol Deviation

Strictly adhere to the protocol approved by the Ethics Committee. Once the researcher identifies any protocol deviation, it should be immediately documented and explained. After review and confirmation by the principal investigator, the information should be submitted to the monitor/sponsor.

3.3 Evaluation

Efficacy Assessment: Evaluate treatment effects by conducting statistical analysis of data collected before and after the intervention.

Safety Assessment

3.3.1 Baseline Signs and Symptoms: Before inclusion in the study, subjects undergo comprehensive inquiry and physical examination. The inclusion and exclusion criteria are strictly followed to ensure subject safety.

3.3.2 Laboratory Safety Assessment: The evaluations in this experiment can be

completed in the ward or outpatient treatment hall. In case of emergencies, timely responses are ensured.

3.3.3 Physical Examination and Vital Signs: All participants in the experiment are medical staff, and subjects must be in good condition during participation. The experiment is conducted within the hospital (ward, outpatient treatment hall). In case of emergencies, medical staff will perform physical examinations and measure vital signs for the subjects.

3.4 Adverse Event Reporting

3.4.1 Adverse Events

Adverse events include: ① Skin tingling sensation in the electrical stimulation area: The patient's experience will be recorded, along with their tolerance level. Generally, the tingling sensation gradually diminishes or disappears after stimulation. If the patient cannot tolerate it, they will withdraw from the experiment. ② Fatigue: Fatigue may occur during exercise therapy or functional assessments. Subjects will be given timely rest, and fatigue levels will be recorded. Fatigue typically alleviates after rest. Throughout the experiment, dedicated medical staff will accompany the patient to ensure their safety as much as possible.

3.4.2 Definition of Adverse Events: Harmful reactions unrelated to the experimental objectives that occur during the normal intervention process.

3.4.3 Severity Assessment: Adverse reactions are categorized into levels 1–5 based on severity:

- Level 1: Mild; asymptomatic or extremely mild symptoms; detectable only through tests; no intervention required.
- Level 2: Moderate; requires minor or non-invasive local intervention; decline in instrumental activities of daily living scale scores.
- Level 3: Severe or clinically significant but not immediately life-threatening; requires hospitalization or extended hospital stay; disability; decline in physical self-care ability

scale scores.

- Level 4: Life-threatening; requires urgent intervention.
- Level 5: Death due to adverse reaction.

3.4.4 Correlation Judgment: Harmful reactions unrelated to the experimental objectives that occur during the experiment, such as skin tingling during electrical stimulation, fatigue during exercise training or functional assessments, or falls, are considered adverse reactions related to this experiment.

4. Ethics

4.1 Ethics Committee

Submit a detailed research plan and related materials to the Ethics Committee.

4.2 Patient Information and Informed Consent

Patient information will be kept strictly confidential. Files will be stored in locked cabinets and accessible only to researchers. Government regulatory authorities or members of the Ethics Review Committee may review your personal data at the research unit as required by regulations. No patient information will be disclosed in the publication of research results. Before participating in the experiment, patients must sign an informed consent form.
